# Sertad1 antagonizes iASPP function by hindering its entrance into nuclei to interact with P53 in leukemic cells

**DOI:** 10.1186/s12885-017-3787-2

**Published:** 2017-11-28

**Authors:** Shaowei Qiu, Shuang Liu, Tengteng Yu, Jing Yu, Min Wang, Qing Rao, Haiyan Xing, Kejing Tang, Yinchang Mi, Jianxiang Wang

**Affiliations:** State Key Laboratory of Experimental Hematology, Institute of Hematology & Blood Diseases Hospital, Chinese Academy of Medical Science & Peking Union Medical College (CAMS & PUMC), 288 Nanjing Road, Tianjin, 300020 People’s Republic of China

**Keywords:** iASPP, Sertad1, P53, Apoptosis, Leukemic cell

## Abstract

**Background:**

As the important suppressor of P53, iASPP is found to be overexpressed in leukemia, and functions as oncogene that inhibited apoptosis of leukemia cells. Sertad1 is identified as one of the proteins that can bind with iASPP in our previous study by two-hybrid screen.

**Methods:**

Co-immunoprecipitation and immunofluorescence were perfomed to identified the interaction between iASPP and Sertad1 protein. Westernblot and Real-time quantitative PCR were used to determine the expression and activation of proteins. Cell proliferation assays, cell cycle and cell apoptosis were examined by flow cytometric analysis.

**Results:**

iASPP combined with Sertad1 in leukemic cell lines and the interaction occurred in the cytoplasm near nuclear membrane. iASPP could interact with Sertad1 through its Cyclin-A, PHD-bromo, C terminal domain, except for S domain. Overexpression of iASPP in leukemic cells resulted in the increased cell proliferation and resistance to apoptosis induced by chemotherapy drugs. While overexpression of iASPP and Sertad1 at the same time could slow down the cell proliferation, lead the cells more vulnerable to the chemotherapy drugs, the resistance to chemotherapeutic drug in iASPP^hi^ leukemic cells was accompanied by Puma protein expression. Excess Sertad1 protein could tether iASPP protein in the cytoplasm, further reduced the binding between iASPP and P53 in the nucleus.

**Conclusions:**

Sertad1 could antagonize iASPP function by hindering its entrance into nuclei to interact with P53 in leukemic cells when iASPP was in the stage of overproduction.

**Electronic supplementary material:**

The online version of this article (10.1186/s12885-017-3787-2) contains supplementary material, which is available to authorized users.

## Background

At present, the incidence of various tumor increased gradually year by year, that had largely threatened the health of human, therefore, lots of researches involved of the pathogenesis and therapy of tumors were performed all over the world. P53, an important tumor suppressor, played an indispensable role in regulation of cell proliferation through induction of growth arrest or apoptosis [1]. Alteration of p53 was frequent in a variety of solid tumors, such as lung, brain. But interestingly, the frequency of that was very low in acute myeloid leukemia (AML), only about 3-8% [2]. But once p53 was mutated or absence in hematological maliganancies, the outcome would be dismal [3, 4].Therefore, it was conceivable that overexpression of oncogenes may be one way to bypass the requirement for p53 mutation in leukemogenesis.

iASPP belonged to the ASPP family consisting of three members, ASPP1, ASPP2 and iASPP. iASPP was described as a shorter protein and identified as a p65 rel A binding protein. iASPP could bind with p53, and prevented it from inducing apoptosis [5–7]. To date, iASPP has been found to be overexpressed in human breast carcinomas, ovarian cancers and so on, it has been confirmed to be related with poor prognosis [8, 9].We had previously detected the expression of iASPP in acute leukemia, and found that the expression of iASPP was significantly higher in patients compared with healthy donors or patients in complete remission [10]. Further we identified a novel isoform of iASPP, named iASPP-SV, and demonstrated that iASPP-SV could inhibit the transactivation of p53 on transcription of its target genes Bax and P21 [11]. By establishing iASPP transgenic mouse model, we found that iASPP could increase the number and reconstitution capacity of hematopoietic stem cells (HSCs), facilitated their resistance to chemotherapy and irradiation [12]. All our previous results suggested that iASPP could play a distinguished role in the pathogenesis of acute leukemia.

To better understand iASPP function and search additional binding partners, the amino terminus of iASPP was used as bait in yeast two-hybrid screen of a cDNA library from human HeLa Matchmaker cDNA library (Clontech). Sertad1 was identified as one of the iASPP binding partners. Sertad1 was known as TRIP-Br1, p34^SEI-1^, positively regulated cell division by binding to cyclin-dependent kinase CDK4. It was also involved in gene transcription, could act as a transcriptional regulator that interacted with the PHD-bromodomain of corepressors and coactivators/adaptor p300/CBP. It possessed transcriptional domains and was differentially overexpressed during the G1 and S phases of the cell cycle [13–15]. Previous studies had shown that Sertad1 was highly expressed in carcinomas from pancreas [16], that considered Sertad1 as an oncoprotein. Hong SW et al. found that Sertad1 could also prevent the ubiquitination and degradation of X-lined inhibitor of apoptosis protein through a direct association, thus, it was suggested that Sertad1 could be a promising target for new antitumor therapy [17].

From the above information, we speculated that the interaction between iASPP and Sertad1 may play a role in the pathogenesis of acute leukemia. In this study, we explored the cell biology of leukemic cell lines when iASPP or Sertad1 was unregulated or downregulated, also binding position and relevant molecular pathways were investigated.

## Results and discussion

### Sertad1 expression level varied in leukemic cell lines and AML patients

**Fig. 1:**
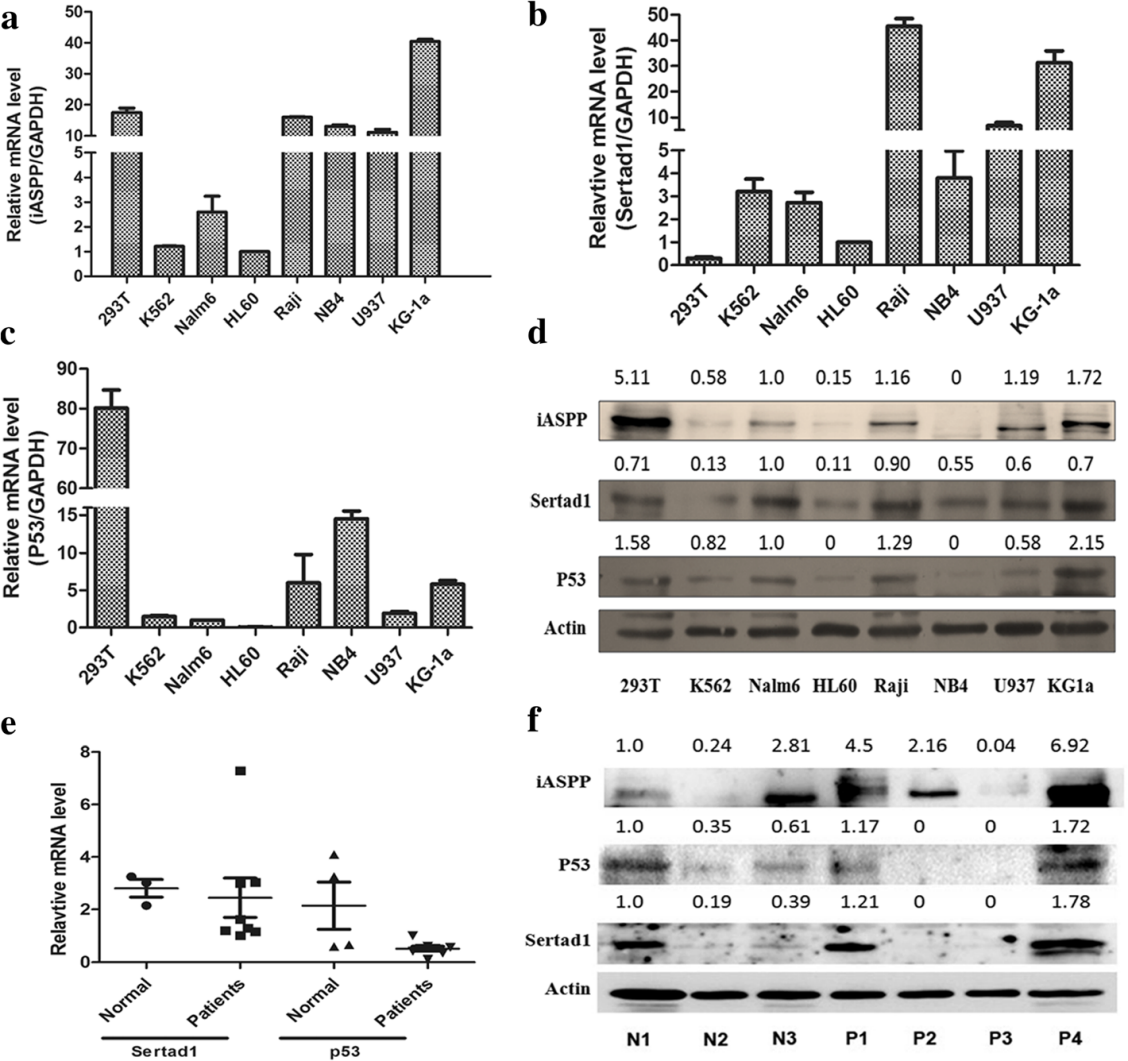
Detection of iASPP, sertad1 and p53 expression in transcriptional and translational level. **a** Expression of iASPP mRNA in 293 T and multiple leukemic cell lines. **b** Expression of sertad1 mRNA in 293 T and multiple leukemic cell lines. **c** Expression of p53 mRNA in 293 T and multiple leukemic cell lines. **d** Expression of iASPP, sertad1 and p53 protein in 293 T and multiple leukemic cell lines. The actin protein was included as a loading control. **e** Expression of sertad1 and p53 mRNA in 4 normal donors (unselected whole bone marrow cells) and 8 primary acute myeloid leukemia patients (the median of the blasts percentage was 82%). GAPDH mRNA was included as control. **f** Expression of iASPP, sertad1 and p53 protein in normal donors(unselected whole bone marrow cells) and primary acute myeloid leukemia patients. N represent normal donor, P represents de novo AML patients. All data are representive of 3 independent experiments

In order to investigate the expression of iASPP and sertad1 in leukemic cells, several leukemic cell lines and bone marrow samples from AML patients were used to analyze the expressions of iASPP and sertad1 at transcriptional and translational levels by real-time RT-PCR and Western blot, and compared with that of 293 T cells. As shown in Fig. 1a, b and c, iASPP and sertad1 were both overexpressed in Raji, NB4, U937 and KG-1a cells, when compared with that in K562, Naml6 and HL60 cells at mRNA level. As the function of iASPP was closely related with P53, the expression of p53 in the above cell lines was also evaluated in Fig. 1d, the protein level of p53 was lower in K562, Nalm6 and HL60 compared with other cells. Integrated with mRNA level and protein level(Fig. 1d), the expression level of iASPP and sertad1 were obviously higher in U937 and KG1a, meanwhile lower in K562, that were further investigated in our study. When we performed the statistic analysis on the correlation of iASPP, Sertad1 and P53 in different cell lines. The data showed that only iASPP was correlated with P53, the Kendall’s tau_b was 0.643(*p* = 0.026) in mRNA level and 0.691(*p* = 0.018) in protein level, but the there was no correlation between Sertad1 and P53 or iASPP and Sertad1.The mRNA and protein level were also explored in primary AML cells, as the Fig. 1e and f implied, the expression level of sertad1 shown no obvious difference between normal donor and AML patients, but the expression level of p53 was lower in AML patients, particular in mRNA level.

### iASPP binds directly to Sertad1 mainly through PHD-bromo domain

**Fig. 2:**
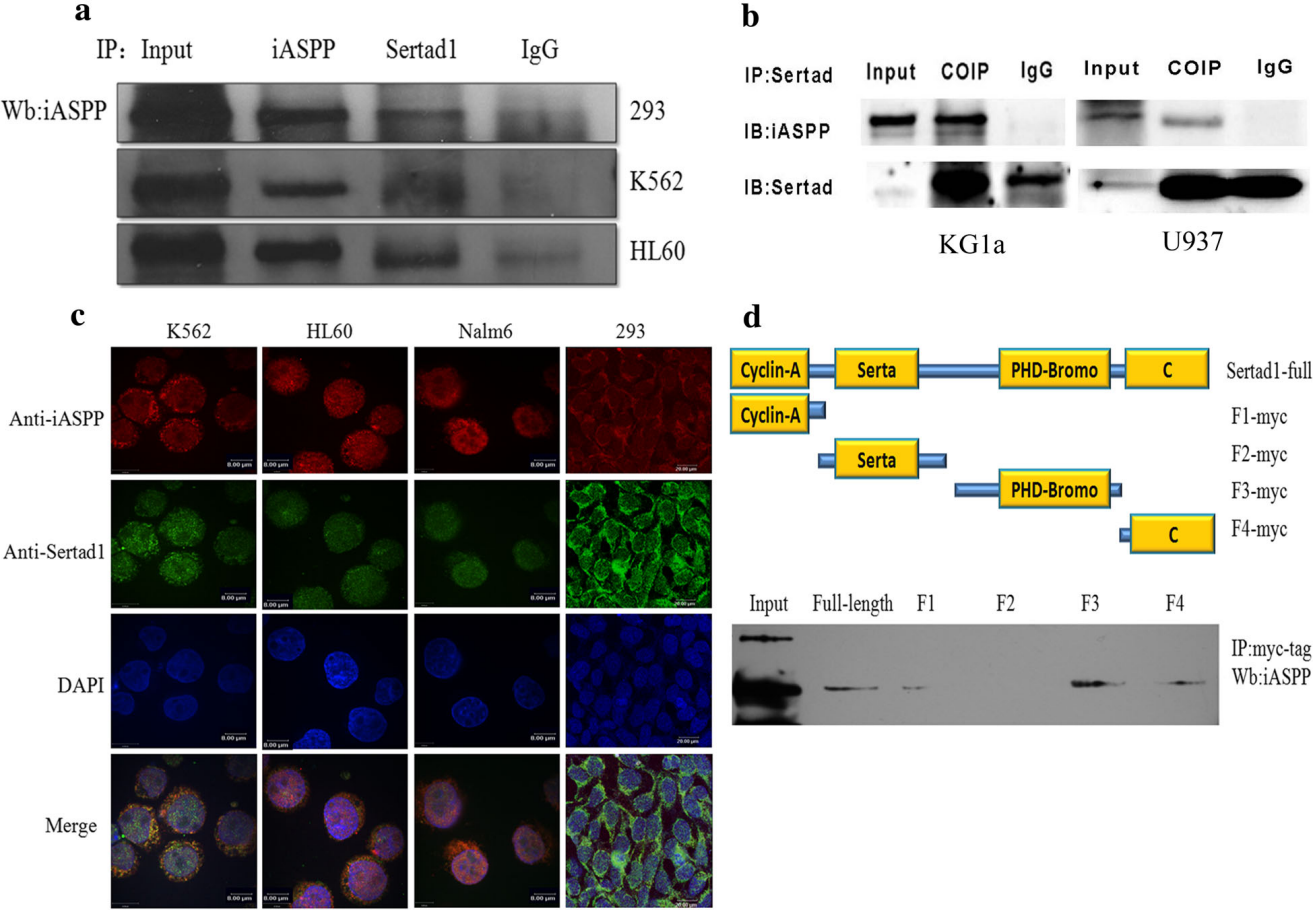
Interaction between iASPP and Sertad1. **a** 293, K562 and HL60 cells were lysed by RIPA lysis buffer, antibody against iASPP and Sertad1 were used for IP-antibody, anti-iASPP antibody was used for Western blot. Input was set as positive control and rabbit IgG antibody for negative control. **b** Co-immunoprecipitation between iASPP and Sertad1 was done in KG1a and U937 cell lines. Antibody against Sertad1 was used for IP, anti-iASPP and anti-Sertad1 were used for IB. **c** Confocal microscopy images of co-localization of iASPP and Sertad1 in K562, HL60, Nalm6 and HEK293 cells by immunostained with antibodies against iASPP(red) and, Sertad1(green), and cell nucleus stained with DAPI (blue). The maximum resolution of the images in K562, HL60 and Nalm6 cells was 8 μm. The maximum resolution of 293 cells was 20 μm. **d** The full-length and different domains of Sertad1 with myc tag were constructed and cotransfected with pcDNA3.1-iASPP into 293 cells. Cell lysates were immunoprecipitated with anti-myc antibody and blotted with anti-iASPP antibody

In our previous experiments, Sertad1 was identified as one of the binding partners of iASPP by yeast two-hybrid screen in vitro. To further confirm the interaction between iASPP and Sertad1, Co-IP was performed in 293 T, K562, HL60 U937 and KG1a. Antibodies against iASPP and Sertad1 were used for IP reaction, and anti-iASPP antibody for WB. The result (Fig. 2a and b) clearly showed that the interaction between iASPP and Sertad1 did exist in 293 cell and leukemic cells. In addition, to better investigate the concrete sub-cellular colocalization of the binding proteins, the fluorescence confocal microscopic imaging of three suspension leukemic cell lines and adherent 293 cell were performed. The results (Fig. 2c) showed that both iASPP and Sertad1 scattered in the cytoplasm and nucleus, and their colocalizations were mainly in the cytoplasm, which encircled the nucleus. Besides the above endogenous colocalization results, the pcDNA3.1-iASPP and pcDNA3.1-Sertad1 plasmids were both transfected into 293 cells at the same time, colocalization of iASPP and Sertad1 was also in the cytoplasm (Data now shown).

The structure of Sertad1 includes four domains, namely Cyclin-A, Serta, PHD-Bromo and C terminal. To understand which domain of Sertad1 can binds directly to iASPP, myc-tagged plasmids which contained the full-length Sertad1, as well as four separated domains of Sertad1 (CylcinA, Serta, PHD-Bromo, C terminal) were constructed, respectively. After co-transfecting 293 cells with each Sertad1 constructs and iASPP, antibodies against myc-tag and iASPP were used for IP and WB, respectively. As shown in Fig. 2d, iASPP binds directly to Sertad1 through its PHD-bromo domain, C-terminal domain and Cyclin-A domain in a reduced order, and Serta domain failed to bind to iASPP.

### Sertad1 antagonizes the function of iASPP

**Fig. 3:**
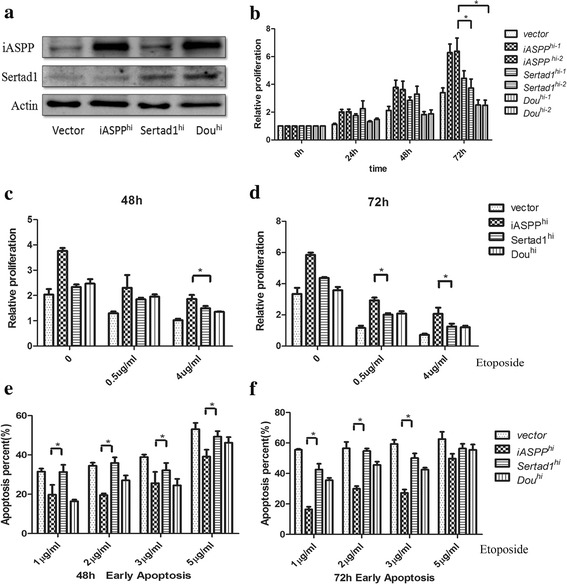
The biological function of iASPP^hi^, Sertad1^hi^ and Dou^hi^ in K562 cells. **a** The protein expression level of iASPP and sertad1 gene in vector, iASPP^hi^, Sertad1^hi^ and Dou^hi^ cells. The actin protein was included as a loading control. **b** The cell proliferation of three groups cells from 0 h to 72 h. **c**-**d** The cell proliferation of three groups after treatment with 0.5 μg/ml and 4.0 μg/ml of etoposide (VP16) for 48 h and 72 h, respectively. **e**-**f** The early apoptosis percentage of three groups after treatment with 1 μg/ml, 2 μg/ml, 3 μg/ml and 5 μg/ml VP16 for 48 h and 72 h, respectively. Early apoptosis represents the population of AnnexinV^+^PI^−^(the apoptosis rate of baseline was below 5%) (* represent *P* < 0.05).All data are representive of 5 independent experiments

To explore the biological functions of iASPP and Sertad1 in the leukemic cell lines, pcDNA3.1-iASPP and pcDNA3.1-Sertad1 were transfected alone or co-transfected into K562 cells, the stable subclones that highly expressed iASPP, Sertad1 or both of them were then established by limiting dilution and named as K562-iASPP^hi^, K562-Sertad1^hi^, and K562-Dou^hi^, respectively. Two subclones of each group were screened by protein for the following function studies, the protein level of each subclone were shown in Fig. 3a.

Firstly, the proliferation of three groups were explored, as shown in Fig. 3b, the proliferation rate of K562-iASPP^hi^ was 2-3 fold higher than that of the control cells, however, the K562-Sertad^hi^ proliferation rate was nearly the same with that of the control cells, interestingly, the proliferation rate of K562-Dou^hi^ cells was lowest, even lower than that of K562-Sertad1^hi^ cells. This finding implied that iASPP overexpression could promote the proliferation of leukemic cell, while Sertad1 overexpression had no impact on the proliferation, but when both iASPP and Sertad1 were up-regulated, the proliferation was slowed down.

Secondly, the anti-apoptosis ability of three groups was further explored. The proliferation of living cells and percentage of apoptosis cells after treatment with chemotherapeutic drugs were detected. Fig. 3c and d showed that K562-iASPP^hi^ cells had greater growth advantage than that of K562-Sertad1^hi^ cells and K562-Dou^hi^ cells when they were treated with 0.5 μg/ml and 4 μg/ml of etoposide for 48 h and 72 h, which implied that iASPP exerted its anti-apoptosis function only in an appropriate stimuli range. Further, we explored the percentage of apoptotic cells in the three groups when treated with etoposide at different concentrations for 48 h or 72 h. From the results of Fig. 3e and f, it was clearly found that the percentage of early apoptosis in K562-iASPP^hi^ cells was lower than other groups in appropriate concentrations (1 μg/ml-3 μg/ml) of VP16, but when the concentration increased to 5 μg/ml, the percentage of early apoptosis in K562-iASPP^hi^ cells was almost the same as other groups. So it is conceivable that iASPP overexpression could exert its anti-apoptotic ability in appropriate ranges. Besides, both the overexpression of iASPP and sertad1 can promote the leukemic cell blocked more in G2/M stage as the Additional file 1: Figure S1 shown. All above results strongly suggested that iASPP could promote the proliferation, inhibit the apoptosis induced by chemotherapy. Sertad1 only affect the cell cycle in leukemic cells, had no effect on proliferation and apoptosis. To our surprise, iASPP was overexpressed in K562-Dou^hi^ cells, but the cell proliferation rate of Dou^hi^ cells was lower than that of control group and the percentage of apoptotic cells was the same with other groups. Thus, it is speculated that the function of iASPP may be antagonized by Sertad1.

### Sertad1 tetherd iASPP protein in the cytoplasm

**Fig. 4:**
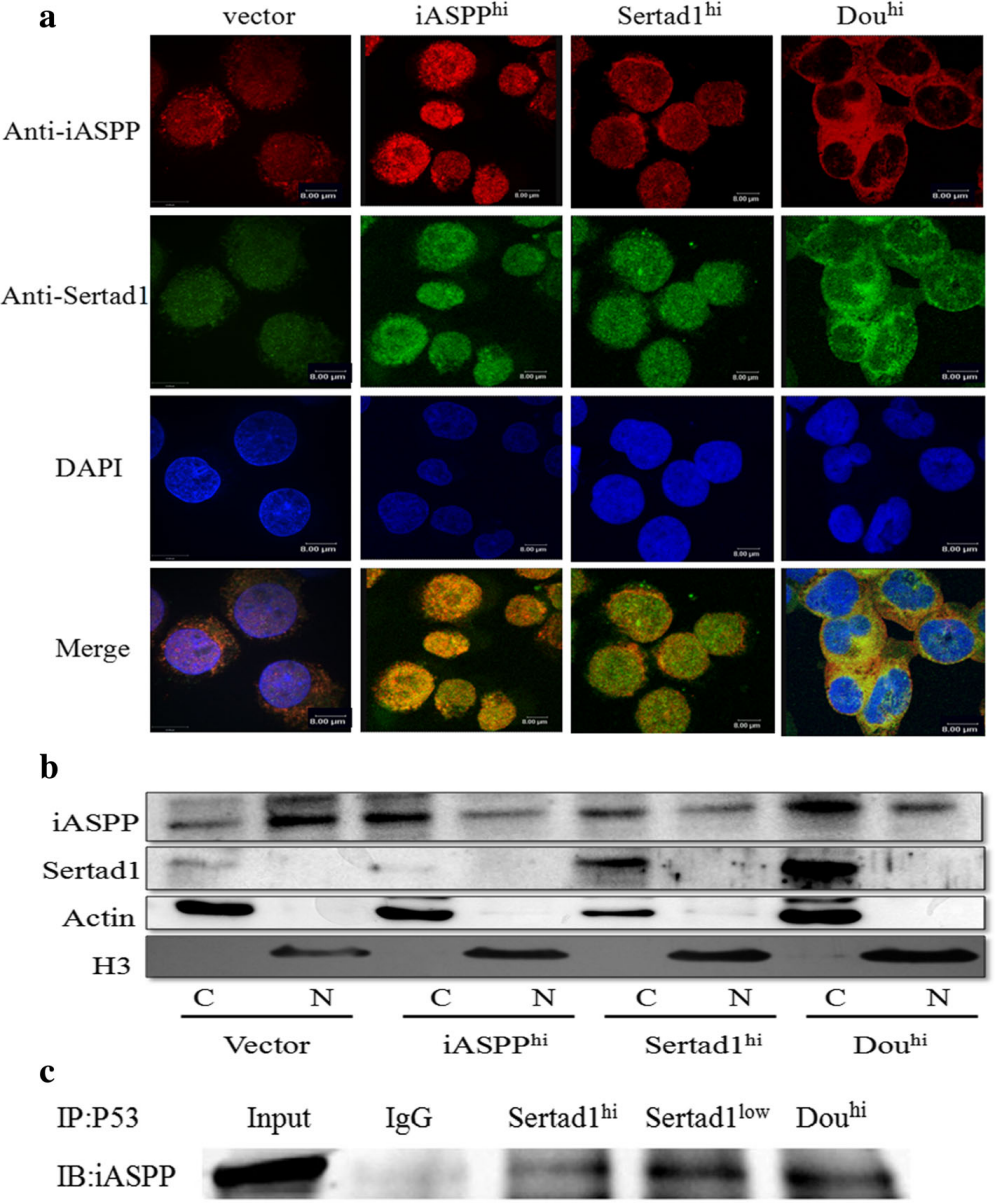
The relocalization of iASPP and Sertad1 in K562-iASPP^hi^, Sertad1^hi^ and Dou^hi^ cells. **a** Confocal microscopy images of relocalization of iASPP and Sertad1 in K562 cells, K562-iASPP^hi^ cells, K562-Sertad1^hi^ cells and K562-Dou^hi^ cells immunolabeled with antibodies against iASPP (red) and Sertad1 (green), and cell nucleus stained with DAPI (blue). The maximum resolution of all the images was 8 μm. **b** The distribution of iASPP and Sertad1 protein in cytoplasm and nucleus. The total protein was lysed by Nuclear and Cytoplasm Protein Kit following the instructions. Actin was used for a loading control of cytoplasm protein, Histone3 for nucleus protein. (C represens cytoplasm protein, N represents nucleus protein). **c** Co-immunoprecipitation between P53 and iASPP was done in Sertad1^hi^, Sertad1^low^ and Dou^hi^ cells. Antibody against P53 was used for IP, anti-iASPP was used for IB

To explore the possible cause of difference between K562-Dou^hi^ cells and K562-iASPP^hi^ cells, the correlation between the subcellular relocalization of iASPP or Sertad1 proteins and this phenomenon was investigated. The subcellular distribution of iASPP and Sertad1 proteins in three transfected K562 cell lines was observed by fluorescence confocal microscopic imaging. As shown in Fig. 4a, in control (vector) and K562-Sertad1^hi^ cells, iASPP and Serad1 proteins scattered mainly in cytoplasm, partly in nuclei, the subcellular colocalization of the two proteins was also outside the nuclei, respectively. In K562-iASPP^hi^ cells, iASPP and Sertad1 scattered diffusely in the cytoplasm and nuclei, the subcellular colocalization of them was not limited to the cytoplasm, also observed in nuclei. But in K562-Dou^hi^ cells, both iASPP and Sertad1 were obviously located in the cytoplasm, which encircled the nuclei, the subcellular colocalization was nearly outside the nuclei. To further confirm the subcellular amount and distribution of iASPP and Sertad1 proteins, the nuclei and cytoplasm protein was extracted separately and analyzed by Western blot. As shown in Fig. 4b, the distribution of iASPP and Setad1 protein was in accord with the results of confocal images, iASPP was mainly located in cytoplasm in Dou^hi^ cells compared with vector cells. Because iASPP exerted its function mainly by P53 protein, the interaction between P53 and iASPP was investigated by immunoprecipitation in Sertad1^hi^,Sertadl^low^ cells, as Fig. 4c shown, the interaction was the lower in Sertad1^hi^ cells compared with Sertadl^low^ cells, that supported the speculation that excess Sertad1 protein could tether iASPP protein in the cytoplasm, further reduced the binding between iASPP and P53 in the nucleus.

### The resistance of iASPP to chemotherapeutic drug was accompanied by puma protein expression in a p53-independent manner

**Fig. 5:**
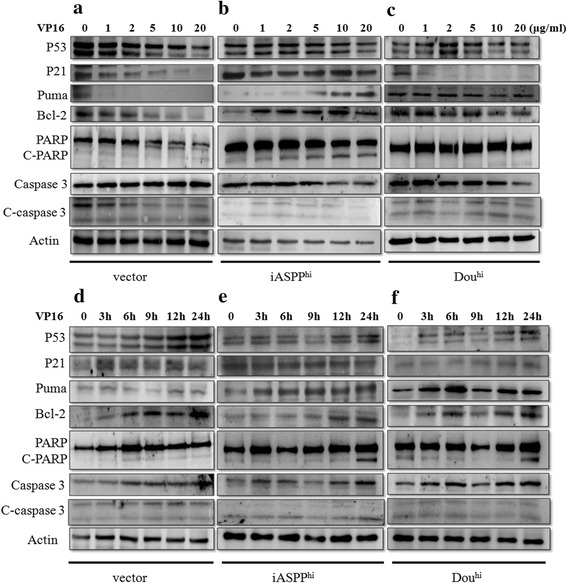
Effects of VP16 on the expression of P53, P21, Puma, Bcl-2, PARP, cleaved-PARP, caspase3 and cleaved-caspases3 in K562, K562-iASPP^hi^ and K562-Dou^hi^ cells. **a**-**c**. K562, K562-iASPP^hi^ and K562-Dou^hi^ cells were treated with 0, 1, 2, 5, 10 and 20 μg/ml of VP16 respectively for 24 h, then the cells were lysed for Western blot analysis. **d**-**f**. K562, K562-iASPP^hi^ and K562-Dou^hi^ cells were treated with 5 μg/ml of VP16 for 0, 3, 6, 9, 12 and 24 h, respectively, then cells were lysed for Western blot analysis. Actin was used for a loading control. C-PARP represents cleaved-PARP, C-caspase3 represents cleaved-caspase3

To better understand the mechanism of anti-apoptosis of K562-iASPP^hi^, P53 and its target proteins including P21, Puma, Bcl-2, PARP, cleaved-PARP, caspase3 and cleaved-caspase3 were assayed to investigate whether they were involved in the apoptotic process. As shown in Fig. 5, K562-vector, K562-iASPP^hi^ and K562-Dou^hi^ cells were exposed to VP16 at different concentrations for 24 h (Fig. 5a-c) or exposed to VP16 at 5 μg/ml for different time (Fig. 5d-f), respectively. As p53 gene was mutated in K562 cells, two bands of P53 with different molecular weights were detected. It was observed that P53 protein decreased gradually as VP16 concentration increased from 0 to 20 μg/ml after 24 h exposure in all the three cell lines (Fig. 5a-c), while P53 protein increased gradually as the exposure time increased from 0 h to 24 h under 5 μg/ml of VP16 treatment (Fig. 5d-f). When looking over the expression trend of p53 target proteins, an interesting phenomenon was observed, namely the Puma protein increased in a time- and dose-dependent manner in VP16 treated K562-iASPP^hi^ cells accompanied by the Bcl-2, cleaved-PARP increment, irrespective of the expression level of P53. But this phenomenon was not observed in K562-vector and K562-Dou^hi^ cells when subjected to the same situation. Because the K562-iASPP^hi^ cells could resist to apoptotic stimuli effectively in a time- and dose-dependent manner (Fig. 3e-f), it suggested that Puma expression might participate in the anti-apoptotic process when considering apoptosis cells decreased obviously in the same situation. As we discussed above, though iASPP exerted its anti-apoptosis ability in some extent, but Puma expression could be activated by the stimuli irrespectively of apoptosis cells number.

### The absence of iASPP had no impact on cell biology

**Fig. 6:**
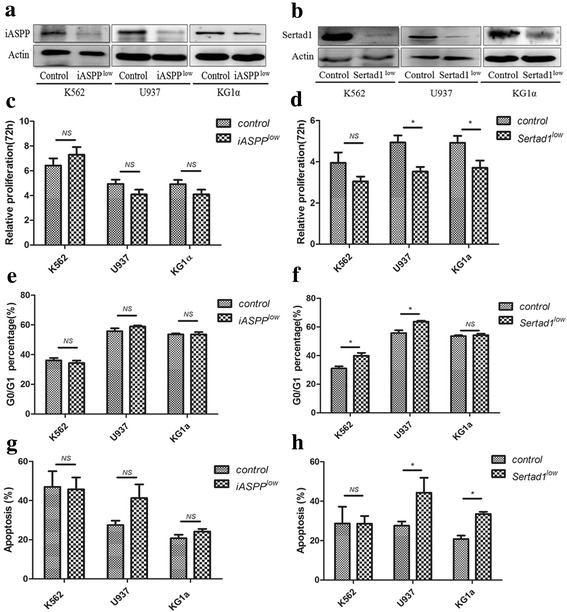
The biological function of the stable clones when iASPP or Sertad1 was knocked down in multiple cell lines. **a**. iASPP was knocked down in the K562,U937 and KG1a leukemic cells. **b**. Sertad1 was knocked down in the K562,U937 and KG1a leukemic cells. **c**. The proliferation of cells after iASPP was knocked down followed by incubated for 72 h in multiple leukemic cells. **d**. The proliferation of cells after Setad1 was knocked down followed by incubated for 72 h in multiple leukemic cells. **e**. The percentage of G0/G1 stage after iASPP was knocked down followed by incubated for 48 h in multiple leukemic cells. **f**. The percentage of G0/G1 stage after Sertad1 was knocked down followed by incubated for 48 h in multiple leukemic cells. **g**. The percentage of apoptosis after iASPP was knocked down followed by incubated for 48 h with etoposide in multiple leukemic cells.(etoptoside in K562 was 2 μg/ml, U937 was 0.4 μg/ml, KG1a was 4 μg/ml). **h**. The percentage of apoptosis after Sertad1 was knocked down followed by incubated for 48 h with etoposide in multiple leukemic cells. (etoptoside in K562 was 2 μg/ml, U937 was 0.4 μg/ml, KG1a was 4 μg/ml).All data are representive of 3 independent experiments. (* represent *P* < 0.05)

As the above results showed that overexprssion of iASPP and Sertad1 could play a role in the biological function of leukemic cells, therefore, silence of the two proteins was performed to further confirm their functions. The expression of iASPP and Sertad1 proteins was knocked down in K562,U937 and KG1a respectively by relevant shRNA(Fig. 6a and b). After that, the cell proliferation, cell cycle and anti-apoptosis ability of above cells were investigated. The results showed that the cell proliferation,cell cycle and cell apoptosis did not change after iASPP was knocked down in all the three leukemic cells (Fig. 6c,e and g).Thus, it is speculated that iASPP is dispensable for maintenance of anti-apoptotic function, cell cycle and cell proliferation. But when Sertad1 was knocked down, to our surprised, the cell proliferation was inhibited(Fig. 6d), the cell cycle were prone to more blocked in G0/G1 stage(Fig. 6f) and leukemic cells became more susceptible to chemotherapy(Fig. 6h). Therefore, the above results suggested the absence of iASPP had no impact on cell biology, but the absence of Sertad1 could change the cell function of leukemic cells, thas was in accord with the oncoprotein function of Seratad1 reported in other solid tumor.

## Conclusions

P53 was one of the most important tumor suppressor genes, it lay at the center of a number of regulatory pathways. iASPP, as the important inhibitor of p53, was found to facilitate cancer progression in more cancer recently [18, 19]. iASPP was considered as an oncogene that not only inhibited the transactivation function of p53 on the promoters by binding with p53, but also promoted carcinogenesis through p53-independent mechanisms [20–22].The overexpression of iASPP in primary mouse embryonic fibroblasts promoted p53 degradation and stimulated cell migration and metastasis [23]. In our study, we found a important partner of iASPP, namely Sertad1. iASPP can bind with Sertad1 protein in multiple leukemic cell lines. When iASPP expression was downregulated by shRNA technology, the biological function of the leukemic cells did not change. When iASPP and Sertad1 were both overexpressed in the leukemic cells, Sertad1 could tether iASPP outside the nucleus, resulting the leukemic cells vulnerable to chemotherapy. It implied that Sertad1 could block iASPP entrance into nucleus when iASPP was in the stage of overproduction.

Because iASPP inhibited the transactivation function of p53 on the promoters by binding with p53, it was conceivable to restore p53 function by relieving the interaction between p53 and iASPP. iASPP silencing by siRNA or shRNA had reduced the proliferation of cancer cells in vitro [24–27]. Some small peptide has been developed to inhibit the apoptotic activity of p53 successfully,such as A34, JNJ-7706621 [18, 28, 29]. Therefore, increasing inhibitors were explored to release p53 from iASPP as the treatment of human tumors through activation of p53 [30]. Our data provided a new insight to inhibt iASPP protein, namely through its binding partner Sertad1, when the similar proteins or drugs that can change the location of iASPP were transfected into the leukemic cells, it may restore p53 function to eliminate the leukemic cells.

Integrating our results, we proposed that in normal situation, the protein iASPP and Sertad1 scattered in both the nucleus and cytoplasm, mainly in the cytoplasm. iASPP could function as oncogene through its binding with P53 protein in the nucleus. When iASPP and Sertad1 were both overexpressed in the leukemic cells, Sertad1 could tether iASPP outside the nucleus mainly through its PHD-bromo domain, reduced the binding between iASPP and P53, eventually prevented iASPP from inhibiting P53 function.

Currently, intensive efforts have been made to restore wildtype p53 activity as an anticancer therapeutic pathway [31].iASPP, as the inhibitor of p53, have arouse enough attentions in the therapeutic target of acute leukemia. Though our study provided valuable evidence about the interaction between iASPP, sertad1 and p53, there were many obstacles awaiting for us to overcome, such as the following fate of iASPP when it was blocked by Sertad1, and whether the impact of Sertad1 on the biology of iASPP could be further confirmed in mouse model. These problems will be the aim of our future study. Above all, the interaction between iASPP and Sertad1 gave us more insights about the regulation of iASPP, including the impact on p53, these results were beneficial to understanding of pathogenesis of acute leukemia and targeted treatment for patients.

## Methods

### Patients

Bone marrow samples from primary acute myeloid leukemia (AML) patients were obtained from Institute of Hematology and Blood Diseases Hospital, Chinese Academy of Medical Sciences and Peking Union Medical College. All patients provided written informed consent for analyses and the investigation was approved by the ethical advisory board of Institute of Hematology and Blood Diseases Hospital. The age range of patients in this study was from 25 to 67. Diagnoses of AML were established according to the criteria of the French-American-British (FAB) co-operative study group.

### Cell culture and plasmid construction

Human leukemic cell lines K562, HL60, U937, KG1a were routinely maintained in RPMI1640 medium supplemented with 10% fetal calf serum (FCS), penicillin (100 U/ml) and streptomycin (100 μg/ml) in a humidified atmosphere of 5% CO_2_ at 37°C. 293 T cells and 293 cells were cultured in Dulbecco’s Modified Eagle Medium (DMEM) supplemented with 10% FCS without antibiotics. The pcDNA3.1-iASPP-V5 plasmid was kindly provided by Prof. Xin Lu from University of Oxford. The pcDNA3.1-p53-Flag plasmid was purchased from Addgene company. Plasmids containing different Sertad1 domains were constructed by PCR and cloned into pcDNA3.1-myc plasmid. iASPP and Sertad1 shRNA fragments were synthesized (Invitrogen) and cloned into PLKO.1 plasmid.

### Cell proliferation assays

The cells were seeded at 10,000 cells per well in a 96-well plates in normal growth medium. 10 μl of 3-(4,5-dimethylthiazol-2-yl)-2,5-diphenyltetrazolium bromide(MTT) reagent was added to each well and incubated for an additional 4 h at 37°C with 5% CO_2_. Media was aspirated and the precipitate was solubilized in 100 μl 10%SDS. The absorbance of each well was measured at 547 nm by Synergy Hybrid Reader (BioTek Instruments, Inc) according to the manufacturer’s instructions. The percentage of viable cells was calculated relative to control wells.

### Cell cycle analysis

The transfected cells were washed twice with PBS and fixed with 70% ethanol for at least 24 h, the cells were then treated with 0.5 mg/ml RNase in 0.1% sodium azide, and incubated at room temperature for 10-15 min followed by staining with 50 μg/ml PI for 10 min. The cells were analyzed for their DNA content using a FACS LSRII flow cytometer (BD Biosciences). Histograms were analyzed for cell-cycle compartments using ModFit version2.0. A minimum of 20,000 cells were collected to maximize statistical validity of the compartmental analysis.

### Apoptosis assessment

The cells were incubated with etoposide (VP16) or adriamycin (ADM) for 48 h and 72 h, washed twice with PBS, and stained with propidium iodide (PI) and annexin V-647 in 100 μl of staining solution at room temperature for 10-15 min in the dark. Samples were then diluted with the binding buffer and analyzed by FACS LSRIIflow cytometer (BD Biosciences) within 0.5 h.

### Co-immunoprecipitation and immunoblotting analysis

To analyze the interaction between iASPP and Sertad1, co-immunoprecipitation (Co-IP) was performed. Briefly, cell lysates were pre-cleared with protein A agarose (Santa Cruz). The supernatants were immunopreciptated with anti-iASPP antibody, followed by incubation with protein A-agarose. Protein A-agarose-antigen-antibody complexes were pelleted by centrifugation. Bound proteins were resolved by SDS-PAGE, followed by Western blot (WB) with antibodies against Sertad1 or myc-tag. The immunoreactive proteins were visualized using SuperSignal chemiluminescent detection system (Pierece).

### RNA interference

Leukemic cell lines were transfected with gene specific shRNA or scrambled shRNA. iASPP shRNA sequence: 5′-CCAACTACTCTATCGTGGATT-3′; iASPP scrambled sequence: 5′-GCACTTAACTCGTAGTCCTAT-3′; Sertad1 shRNA sequence: 5′- AACGGGTCTGAAGGGAAACGG-3′; Sertad1 scrambled sequence: 5′- GAGCGA- GTGCAACGAGAGAGT-3′. All above PLKO.1-shRNA plasmids were constructed in our laboratory. The cell lines expressing shRNAs were maintained in 0.8 μg/ml puromycin.

### Immunofluorescence

For co-localization of iASPP and Sertad1, immunofluorescence staining was performed. The cells were subjected to two washes in PBS, and then were fixed with 4% paraformaldehyde for 15 min, permeabilized with 0.25% Triton X-100 in PBS for 10 min and blocked with 2% horse serum for 30 min at room temperature. Cells were then incubated with primary antibody diluted at 1:100 at 4°C overnight. After three washes in PBS, cells were incubated with DyLight™ 448 donkey anti-rabbit IgG or DyLight™ 649 goat anti-mouse IgG diluted at 1:100 for 1 h at room temperature, DAPI was used for nuclear staining. Observations were made using Leica TCS P2 microscope.

### RNA isolation, RT-PCR and RQ-PCR

iASPP and Sertad1 mRNA were assessed by RT-PCR or RQ-PCR. Total RNA from de novo AML samples and leukemic cell lines were isolated, cDNA was synthesized using a reverse transcription kit (Invitrogen). Primers used for PCR were listed as follows:(1) iASPP 5′-CGCGGGACTTTCTGGACATG-3′ and 5′- TGCCGAAGGGC TCAGGAATC-3′ (2)Sertad1 5′-CTCATGGATGTGCTGGTGG-3′ and 5′-AGGAC GGATGTGAAGTTGC-3′.

### Statistical analysis

Statistical difference between experimental groups was calculated and analyzed using Student’s t test. All experiments were performed in triplicate and averaged from >3 independent experiments. All tests were two-tailed and considered significant at a *p* < 0.05. All calculations were performed with SPSS version13.0.

## Additional file

Additional file 1: Figure S1. The percentage of cell cycle in G0/G1, S and G2/M in K562-iASPP^hi^, Sertad1^hi^ and Dou^hi^ cells. (ZIP 2313 kb)

## Abbreviations

ADM: Adriamycin
CO-IP: Co-immunoprecipitation
PR-PCR: Real-time quantitative polymerase chain reaction
RT-PCR: Reverse transcription-polymerase chain reaction
VP16: Etoposide
WB: Western blot
