# Supplementary material for: Sertad1 antagonizes iASPP function by hindering its entrance into nuclei to interact with P53 in leukemic cells
Source: BMC Cancer. 2017 Nov 28;17:795. doi: 10.1186/s12885-017-3787-2 (PMC5704379; doi:10.1186/s12885-017-3787-2)
Supplement: Additional file 1: Figure S1. — The percentage of cell cycle in G0/G1, S and G2/M in K562-iASPPhi, Sertad1hi and Douhi cells. (ZIP 2313 kb) [file 12885_2017_3787_MOESM1_ESM.zip › Supplemental Figure LegendR2.docx]

**Additional file 1: Figure S1.**

The percentage of cell cycle in G0/G1, S and G2/M in K562-iASPP^hi^, Sertad1^hi^ and Dou^hi^ cells
