# Supplementary figures and images for: Sertad1 antagonizes iASPP function by hindering its entrance into nuclei to interact with P53 in leukemic cells
Source: BMC Cancer. 2017 Nov 28;17:795. doi: 10.1186/s12885-017-3787-2 (PMC5704379; doi:10.1186/s12885-017-3787-2)

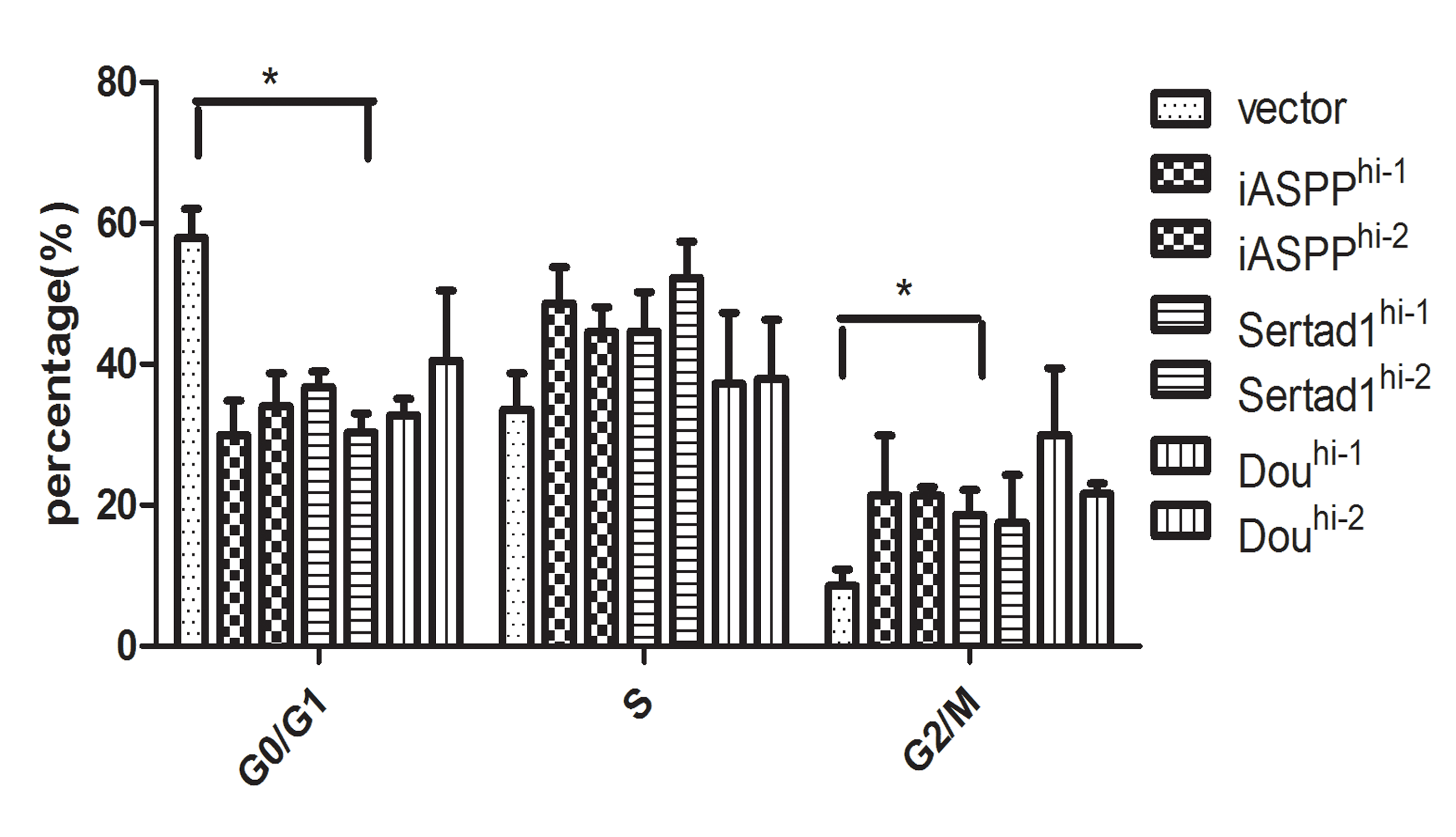

Supplement: Additional file 1: Figure S1. — The percentage of cell cycle in G0/G1, S and G2/M in K562-iASPPhi, Sertad1hi and Douhi cells. (ZIP 2313 kb) [file 12885_2017_3787_MOESM1_ESM.zip › FigS1R2.tif]
